# Investigation of SNPs in GDF9 gene and their relationship with some reproductive traits of Ossimi and Rahmani sheep in different lambing seasons

**DOI:** 10.1186/s12917-025-05145-5

**Published:** 2025-12-02

**Authors:** Mahmoud H. Hasanain, Ahmed S. A. Sosa, Hassan R. H. Darwish, Abdelaziz M. Sakr, Sameh M. Shedeed

**Affiliations:** 1 Department of Animal Reproduction & AI, Veterinary Research Institute, National Research Centre, Dokki, Giza, 12622 Egypt; 2 Cell Biology Department, Institute of Biotechnology Research, National Research Centre, Dokki, Giza, 12622 Egypt; 3 Animal Production Research Institute, Agriculture Research Centre, Ministry of Agriculture, Dokki, Giza, 12619 Egypt

**Keywords:** GDF9 gene, Polymorphisms, Lambing season, Reproductive traits, Ossimi and rahmani sheep

## Abstract

**Background:**

Fertility is an important economic trait for sheep breeding. The growth differentiation factor 9 (GDF9) is a key gene influencing the reproductive traits in sheep. We focused on investigating SNPs in the GDF9 gene and their relationship with some reproductive traits like pre-mating weight, lambing weight, litter size (LS), DRIL (days from rams’ introduction to lambing), lamb birth weight, lamb gender and survival rate in Ossimi and Rahmani sheep. PCR, Single‑strand conformational polymorphism and sequencing were performed on the genomic DNA of 231 ewes (144 Ossimi and 87 Rahmani) to demonstrate polymorphisms in a fragment of 274 bp within exon 2 of the GDF9 gene.

**Results:**

Two linked SNPs (A/G and G/A) were observed in GDF9 gene in Ossimi and Rahmani sheep, revealed by two haplotypes (AAGG and AGGA). For Rahmani sheep, the haplotype AGGA was significantly (*P* value < 0.001) higher than AAGG in lambing weight and LS (1.40 ± 0.02) but AAGG was markedly higher in pre-mating weight and lamb birth weight. In Ossimi sheep, the AGGA haplotype was significantly (*P* value < 0.0001) greater in pre-mating weight, lambing weight and lamb birth weight than AAGG. The effect of the interaction of GDF9 gene polymorphism and the lambing season showed that in Rahmani sheep AGGA haplotype in winter was significantly (*P* value is < 0.0001) increased in pre-mating weight and LS (1.80 ± 0.03) than other interactions but it was significantly (*P* value < 0.0001) higher for lambing weight and lower in DRIL (159.0 ± 1.0) in spring than other seasons. In Ossimi sheep AGGA haplotype in spring was significantly higher in pre-mating weight, lambing weight, LS (1.50 ± 0.07) and lamb birth weight but lower in DRIL (163.17 ± 2.71 days) than in other seasons. The ewes with the AAGG haplotype gave birth to more males (56.3%) than the heterozygous ones (33.3%) in Ossimi sheep, unlike Rahmani.

**Conclusion:**

SNPs in the GDF9 gene can be used as potential genetic markers for reproductive traits in Ossimi and Rahmani sheep where AGGA can be the desirable haplotype for litter size, DRIL, lamb birth weight, ewe pre-mating weight and ewe lambing weight.

**Supplementary Information:**

The online version contains supplementary material available at 10.1186/s12917-025-05145-5.

## Background

 Achieving the economic and productive efficiency goals in sheep farms is directly associated with their reproductive management. Litter size is a crucial reproductive trait that improve productivity of sheep through increasing number of lambs, meat and wool [1]. It is defined as the total number of lambs born per ewes lambing [2]. There is a great variation in the litter size in different breeds of sheep over the world as it is determined by genetics and the environment with a moderate heritability [3]. Most of the breeds produce a single lamb per pregnancy while few breeds produce twins or triplets [4]. This is usually related to the rate of ovulation of the ewes [5, 6]. By the way, the ovulation rate in sheep is affected by the genetics, age, breeding season and nutrition of the animal [7]. Lamb birth weight is another essential parameter in lamb production evaluation criteria in sheep [8]. It affects the development and mortality rates of lambs [9]. Also, lamb survival rate is a very crucial factor for highly prolificacy in sheep production [10]. It is defined as the number of survived lambs after birth to the total number of born lambs. It is higher in newborn lambs of ewes with greater pre-mating weight [11, 12]. So, evaluating the pre-mating weight of ewes is of great interest for the reproductive performance of ewes [13]. It is positively associated with their ovulation rates [14]. The increase in premating weights of the ewes has led to subsequent increases in multiple birth, lamb birth weight and lamb weaning weight [11, 12].

Understanding the genetic background of the reproduction traits is essential to improve the productivity in sheep farms [15]. Hence, selection and breeding studies utilizing molecular techniques play a crucial role in the genetic improvements for reproductive efficiency in sheep [16]. In this context, fecundity trait in sheep is genetically regulated by various major genes associated with litter size and ovulation rate called fecundity (Fec) genes [17]. Growth differentiation factor 9 (GDF9) gene is one of these Fec genes known as FecG. It is a member of the transforming growth factor β superfamily associated with folliculogenesis and female fertility. GDF9 is derived from oocytes and it is important for follicular development and ovulation in sheep [18]. The gene is located on sheep chromosome 5 and spans about 2.5 kilobases (kb) containing two exons separated by an intron with the length of 1126 base pairs [19, 20]. The long-mature peptide of this gene consists of 135 amino acids, while the prepropeptide has 453 amino acids [21]. Mutations in the GDF9 gene can enhance ovarian activity by increasing ovulation rates of ewes [22]. So, this gene has major effects on litter size in sheep [5, 23, 24]. GDF9 is derived from oocytes and contributes to sheep reproduction, including follicular development, oogenesis, and ovulation [25, 26], as it affects transmembrane receptor-protein serine/threonine kinases and is involved in ovulation cycles, gamete production, and gonadal development [27]. SNPs in the GDF9 gene have been shown to influence the fertility traits of sheep. There have been several studies on polymorphisms of GDF9 gene and their relation with litter size in various sheep breeds [24, 25, 28–30]. Hence, studies on the GDF9 can provide useful information for marker-assisted breeding to predict sheep fertility traits and increase litter size. So, this work aimed to investigate the single nucleotide polymorphisms in exon 2 of the GDF9 gene and their association with some reproductive traits such as ewe pre-mating weight, ewe lambing weight, litter size, DRIL (days from rams’ introduction to lambing), lamb birth weight, lamb gender and survival rate in Ossimi and Rahmani Egyptian sheep breeds in different lambing seasons.

## Results

### The observed SNPs and haplotypes of GDF9 gene

**Fig. 1:**
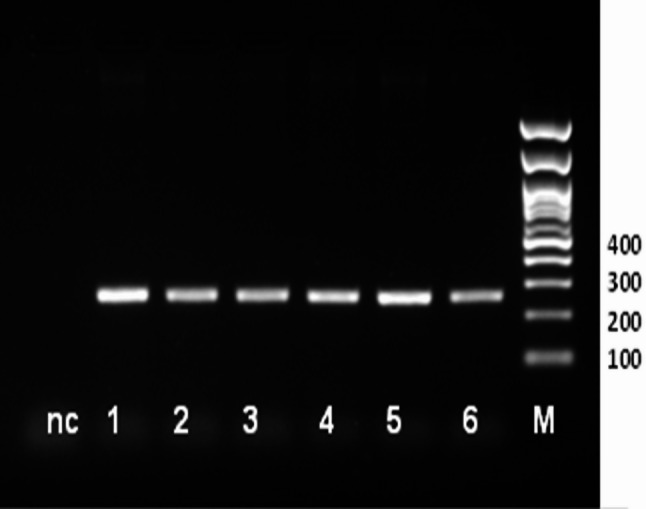
274 bp PCR amplified fragment of GDF9 gene on 1.5% agarose gel. nc: negative control, Lanes 1–6 for PCR bands, M: 100 bp. DNA marker

**Fig. 2:**
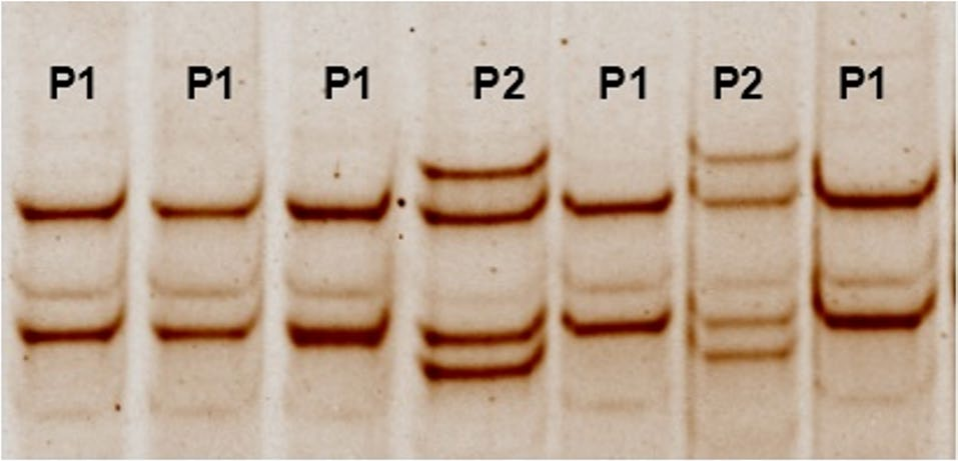
SSCP of 274 bp PCR fragment in GDF9 gene on 10% polyacrylamide gel, P; polymorphic patterns 1 and 2

**Fig. 3:**
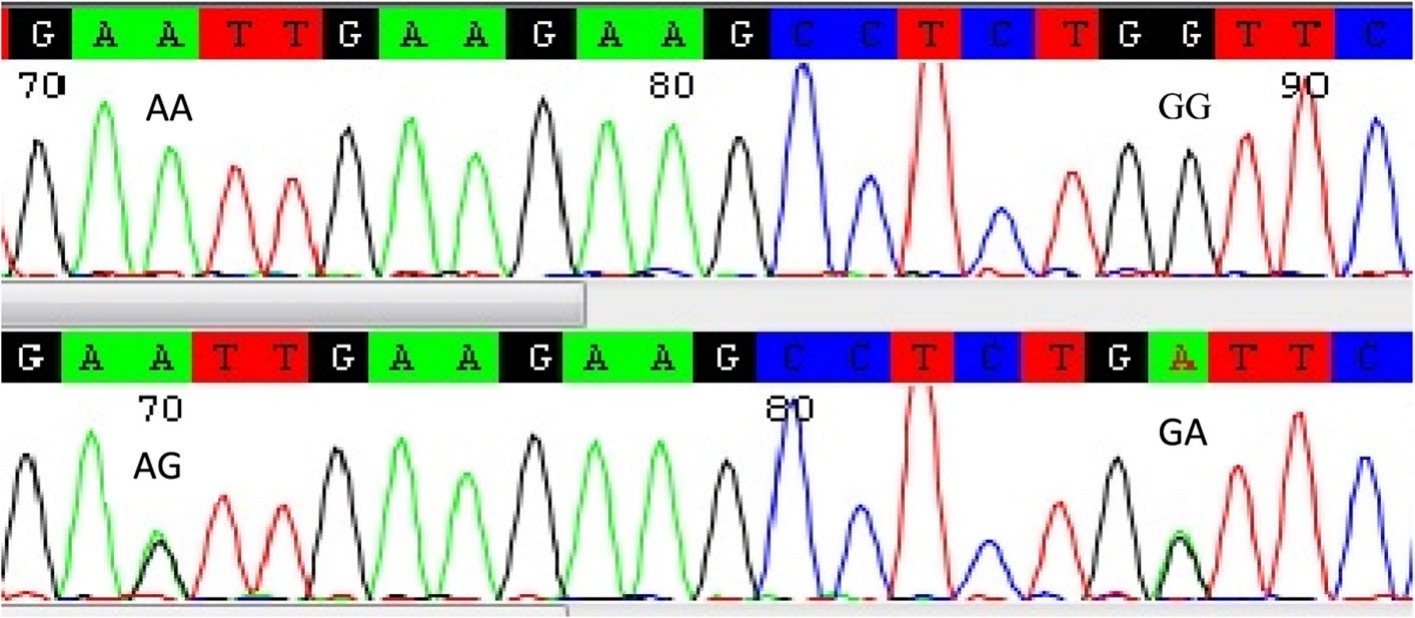
Analysis of partial sequence GDF9 gene by CodonCode Aligner showing two SNPs (A/G and G/A) represented by 2 haplotypes; AAGG and AGGA

**Fig. 4:**
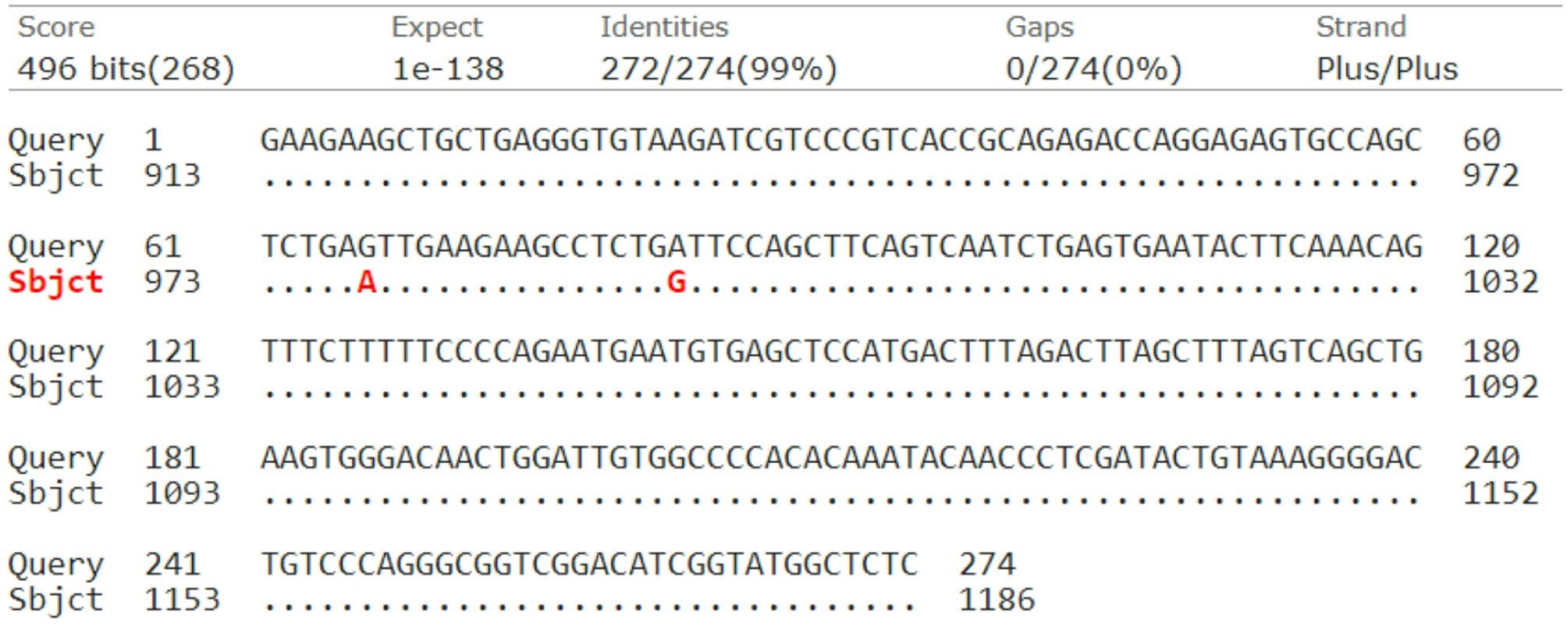
The nucleotide sequence alignment of 274 bp in GDF9 gene by NCBI/BLAST/blastn suite, with the sequence NM_001142888.2 in the GenBank showed 2 SNPs (N66: A/G and N82: G/A)

**Fig. 5:**
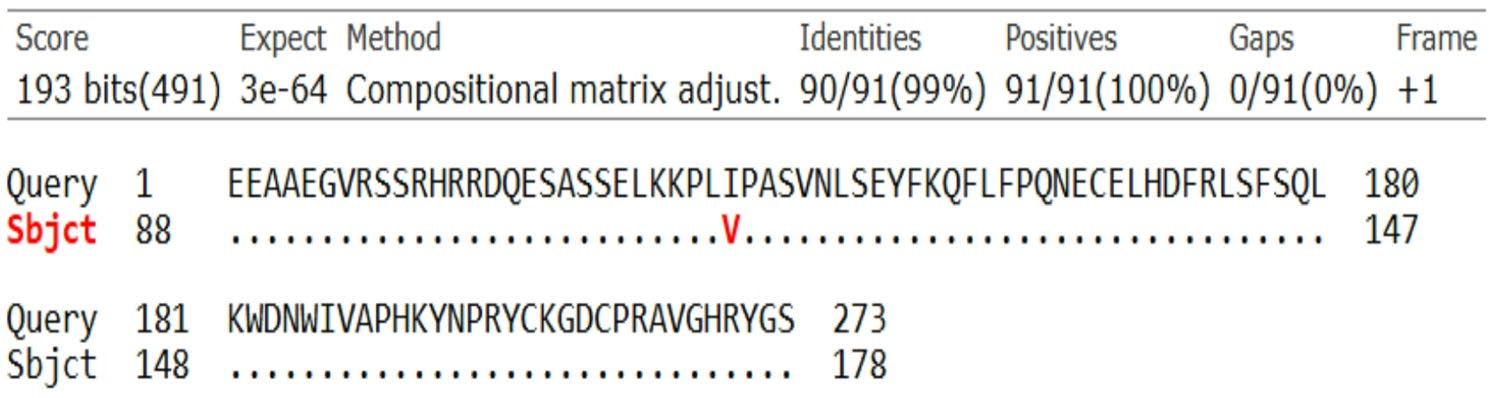
The amino acid sequence alignment of 274 bp fragment in GDF9 gene by NCBI/BLAST/blastx suite, with the sequence in the GenBank showing 1 amino acid change (V/I)

PCR technique was carried out on the genomic DNA of studied Ossimi and Rahmani sheep and amplified a fragment of 274 bp in exon *2* of the GDF9 gene (Fig. 1). When SSCP technique was performed on the PCR products, two different band patterns were observed (Fig. 2). The nucleotide sequencing demonstrated 2 SNPs (A/G and G/A) revealed by two genotypes in the two loci (AA and AG genotype for the first locus and GG and GA genotype for the second locus) in both Ossimi and Rahmani sheep (Fig. 3). By observing the genotypes of the two loci, tight linkage exists between the two SNPs utilizing two haplotypes; AAGG for homozygous and AGGA for heterozygous patterns. The sequence alignment of the 274 bp fragment with sequence ID: NM_001142888.2 and AF078545.2 for *Ovis aries GDF9* gene in the GenBank showed a transition single base substitution in the nucleotide No. 66 (A > G) and 82 (G > A) in the haplotype AGGA (Fig. 4) while the sequence for AAGG haplotype was 100% identical with these sequences. This was further confirmed using Genome assembly ARS-UI_Ramb_v3.0 GCF_016772045.2; reference was 5: 42,114,301–42,114,574 and SNPs were located 5: 42,114,509 (G/A) and 5: 42,114,493 (A/G). The sequence data of haplotype AGGA were submitted to the GenBank and got accession number; PV089625.1. The sequence alignment of the amplified fragment with the GDF9 amino acids sequence in the GenBank revealed 1 amino acid change (V/I) for the second mutation (G/A) at residue 332 but the first locus showed silent synonymous mutation (Fig. 5). The frequencies of the haplotypes of GDF9 gene represented low heterozygousity (AGGA) in Ossimi (18.8%) and Rahmani (10.3%) sheep with no significant difference in between (Table 1).

**Table 1:** Haplotype frequencies of GDF9 gene in ossimi and Rahmani sheep populations

Breed * Haplotype			Haplotype		*P* value
			AAGG	AGGA	
Breed	Ossimi	Count	117	27	0.088
		% within Breed	81.3%	18.8%	
	Rahmani	Count	78	9	
		% within Breed	89.7%	10.3%	

Pearson Chi-Square

### Least square means± standard error of means (LSM± SEM) of some reproductive traits for different GDF9 polymorphisms and lambing seasons in Rahmani sheep

Table 2 showed the LSM ± SEM of ewe pre-mating weight, ewe lambing weight, litter size, DRIL (days from rams’ introduction into the flock till ewe lambing) and lamb birth weight for the observed GDF9 haplotypes in Rahmani sheep. There were significant differences (*P* value is < 0.001) where the heterozygous haplotype AAGG was significantly higher in ewe pre-mating weight (41.26 ± 0.18 kg) and lamb birth weight (3.22 ± 0.04 kg) than AGGA. In contrast, AGGA haplotype was significantly increased in ewe lambing weight (41.37 ± 0.15 kg) and litter size (1.40 ± 0.02) than AAGG. DRIL revealed non-significant difference between the two haplotypes. The effects of interaction of GDF9 haplotypes and lambing seasons on the reproductive traits in Rahmani sheep were presented in Table 3. The haplotype AGGA in winter was significantly (*P* value is < 0.0001) higher in ewe pre-mating weight (42.70 ± 0.20) and litter size (1.80 ± 0.03) than other interactions (AAGG*winter, AAGG*spring, AAGG*autumn, AGGA*spring and AGGA*autumn). Also AGGA in spring was significantly (*P* value is < 0.0001) higher in ewe lambing weight (45.00 ± 0.27) and lower in DRIL (159.0 ± 1.0) than other interactions. But AGGA in autumn was significantly (*P* value is < 0.003) lower in lamb birth weight (2.50 ± 0.07 kg) than other interactions.

**Table 2:** LSM ± SEM of some reproductive traits for haplotypes of GDF9 in Rahmani sheep

Variance of difference	Ewe pre-mating weight(Kg)	Ewe lambing weight (Kg)	DRIL (days)	litter size (No.)	litter size (No.)	Lamb birth weight (Kg)	Lambbirth weight (Kg)
Haplotype							
AAGG	41.26 ± 0.18^a^	40.19 ± 0.17^b^	180.9 ± 0.6^a^		1.30 ± 0.02^b^		3.22 ± 0.04^a^
AGGA	40.32 ± 0.15^b^	41.37 ± 0.15^a^	178.7 ± 0.5^a^		1.40 ± 0.02^a^		2.93 ± 0.03^b^
*P* value	< 0.001	< 0.001	0.12		< 0.001		< 0.001

Values within the same column with different superscript letters are significantly different

**Table 3:** Effects of interaction of GDF9 polymorphisms and lambing seasons on reproductive traits in Rahmani sheep (LS means ± S.E.)

Variance of difference	Ewe pre-mating weight(Kg)	Ewe lambing weight (Kg)	DRIL (Days)	Litter size (No.)	Lamb birth weight (Kg)
Haplotype*Lambing season					
AGGA*winter	42.70 ± 0.20^a^	40.60 ± 0.19^b^	165.7 ± 0.7^d^	1.80 ± 0.03^a^	3.30 ± 0.04^a^
AAGG*winter	41.47 ± 0.27^b^	40.94 ± 0.26^b^	169.0 ± 0.9^c^	1.31 ± 0.04^c^	3.41 ± 0.06^a^
AAGG*spring	41.13 ± 0.38^b^	40.19 ± 0.37^bc^	171.1 ± 1.3^c^	1.44 ± 0.05^b^	3.25 ± 0.09^a^
AAGG*autumn	41.18 ± 0.25^b^	39.45 ± 0.24^c^	202.6 ± 0.8^b^	1.16 ± 0.03^d^	3.00 ± 0.06^b^
AGGA*spring	38.00 ± 0.28^d^	45.00 ± 0.27^a^	159.0 ± 1.0^e^	1.40 ± 0.04^bc^	3.00 ± 0.06^b^
AGGA*autumn	40.25 ± 0.31^c^	38.50 ± 0.31^d^	211.5 ± 1.1^a^	1.0 ± 0.04^e^	2.50 ± 0.07^c^
*P* value	< 0.0001	< 0.0001	< 0.0001	< 0.0001	0.003

Values within the same column with different superscript letters are significantly different

#### Effects of GDF9 polymorphisms and lambing seasons on reproductive traits in Ossimi sheep

Table 4 represented the LS means ± S.E. of the reproductive traits for the different haplotypes of GDF9 gene in Ossimi sheep. The haplotype AGGA was significantly higher than AAGG in ewe pre-mating weight (*P* value < 0.0001), ewe lambing weight (*P* value < 0.0001) and lamb birth weight (*P* value is 0.004). But DRIL and litter size showed no significant differences between the two haplotypes. The effects of interactions of GDF9 polymorphisms and lambing seasons on the inspected reproductive traits in Ossimi sheep were discussed in Table 5. Briefly, the haplotype AGGA in spring was significantly greater in ewe pre-mating weight (*P* value < 0.0001), ewe lambing weight (*P* value < 0.0001) and litter size (1.50 ± 0.07, *P* value is 0.05) but lower for DRIL (163.17 ± 2.71 days, *P* value is 0.02) than other interactions of GDF9 haplotype and lambing season. Also the haplotype AGGA in spring and winter were significantly higher for lamb birth weight (*P* value = 0.024) than other interactions.

**Table 4:** LS means ± S.E. of the reproductive traits for the haplotypes of GDF9 in Ossimi sheep

Variance of difference	Ewe pre-mating weight(Kg)	Ewe lambing weight (Kg)	DRIL (Days)	litter size (No.)	lamb birth weight (Kg)
Haplotype					
AGGA	41.76± 0.33^a^	40.76± 0.33^a^	181.2± 2.1^a^	1.24± 0.04^a^	3.38 ± 0.07^a^
AAGG	39.71± 0.15^b^	39.04± 0.15^b^	184.0± 0.9^a^	1.23± 0.02^a^	3.17± 0.03^b^
* P* value	< 0.0001	< 0.0001	0.224	0.859	0.004

Values within the same column with different superscript letters are significantly different

**Table 5:** Effects of interaction of GDF9 polymorphisms and lambing season on some reproductive traits in Ossimi sheep (LS means ± S.E.)

Variance of difference	Pre-mating weight(Kg)	Ewe lambing weight (Kg)	DRIL (Days)	Litter size (No.)	Lamb birth weight (Kg)
Haplotype*Lambing season					
AGGA*spring	44.17± 0.62^a^	43.33± 0.60^a^	163.2± 2.8^c^	1.50± 0.07^a^	3.58± 0.06^a^
AAGG*spring	39.54± 0.29^b^	39.79± 0.28^b^	169.5± 1.3^b^	1.36 ± 0.03^b^	3.36± 0.06^b^
AGGA*autumn	40.33± 0.50^b^	39.22± 0.49^b^	199.0± 2.2^a^	1.22± 0.06^bc^	3.03± 0.07^c^
AAGG*winter	40.37± 0.25^b^	39.22± 0.25^b^	168.5± 1.1^b^	1.20± 0.03^bc^	3.23± 0.10^bc^
AGGA*winter	40.47± 0.25^b^	39.42±0.25^b^	168.5± 1.1^b^	1.22± 0.03^bc^	3.67± 0.05^a^
AAGG*autumn	39.46± 0.22^b^	38.63±0.22^b^	203.4± 1.0^a^	1.13± 0.03^c^	3.01± 0.06c
*P* value	< 0.0001	< 0.0001	0.020	0.05	0.024

Values within the same column with different superscript letters are significantly different

#### Effect of ewe GDF9 haplotypes on lamb gender and survival rate in Ossimi and Rahmani sheep

Table 6 showed the frequencies of male and female born lambs in different GDF9 haplotypes of ewes in Ossimi sheep. There was significant difference (*P* value < 0.001) in the gender of the new born lambs where the ewes with homozygous haplotype (AAGG) gave birth of more males (56.3%) than the heterozygous ones (33.3%). In Rahmani sheep, there were no significant differences (*P* value = 0.3) between the two GDF9 haplotypes in the gender of the new born lambs (Table 7). Both haplotypes gave birth of fewer male lambs (40.2% and 33.3% respectively) than female ones. Table 8 summarized the frequencies of live and dead born lambs in relation to GDF9 haplotypes of their mothers in Ossimi sheep. No significant difference was obvious in the lamb survival rate between the two haplotypes. Also in Rahmani sheep, no significant variation (*P* value = 0.882) between the two GDF9 haplotypes in the survival rate of lambs (Table 9).

**Table 6:** Frequencies of male and female born lambs in relation to ewe GDF9 haplotypes for Ossimi sheep

Haplotype* gender			Male	Female	Total
Haplotype	AAGG	Count	252	196	448
		% within haplotype	56.3%	43.8%	100.0%
	AGGA	Count	28	56	84
		% within haplotype	33.3%	66.7%	100.0%

Pearson Chi-square < 0.001

**Table 7:** Frequencies of male and female born lambs in relation to ewe GDF9 haplotypes in Rahmani sheep

Haplotype* gender			Male	Female	Total
Haplotype	AAGG	Count	140	208	348
		% within haplotype	40.2%	59.8%	100.0%
	AGGA	Count	20	40	60
		% within haplotype	33.3%	66.7%	100.0%

Pearson Chi-square = 0.3

**Table 8:** Frequencies of lambs survival in relation to ewe GDF9 haplotypes in Ossimi sheep

Haplotype* lamb survival			Dead	Live	Total
Haplotype	AAGG	Count	40	408	448
		% within haplotype	8.9%	91.1%	100.0%
	AGGA	Count	8	76	84
		% within haplotype	9.5%	90.5%	100.0%

Pearson Chi-square = 0.861

**Table 9:** Frequencies of lambs survival in relation to ewe GDF9 haplotypes in Rahmani sheep

Haplotype* lamb survival			Dead	Live	Total
Haplotype	AAGG	Count	44	304	348
		% within haplotype	12.6%	87.4%	100.0%
	AGGA	Count	8	52	60
		% within haplotype	13.3%	86.7%	100.0%

Pearson Chi-square = 0.882

## Discussion

GDF9 gene is one of the major genes affecting reproductive traits in sheep. In the current research, we analyzed the polymorphic variations of GDF9 gene in Ossimi and Rahmani sheep; local Egyptian sheep breeds. Using the SSCP technique and sequence analysis, two SNPs were observed (A >G and G >A). The sequence alignment showed 1 amino acid change (V/I) at residue 332 due to the second mutation G >A. This finding was in accordance with Aymaz, ÖZDİL [31] who mentioned that the G >A is a missense mutation affecting the mature GDF9 protein as it was predicted to change the tertiary structure of that protein. The first SNP was a transition mutation (A >G). The frequencies of both SNPs were identical concluded that the two SNPs were tightly linked to each other. The two haplotypes in the GDF9 gene were observed in both Rahmani and Ossimi sheep but different in frequencies. The presence of the two haplotypes in our findings explained that the gene is not conserved in Ossimi and Rahmani sheep breeds. The frequency of heterozygous haplotype (AGGA) was higher in Ossimi sheep. This was in accordance with Aymaz, ÖZDİL [31] for Chios sheep. While the heterozygosity was lower in Rahmani sheep. Previously, these heterozygosity were lower in case of Wadi and Hu sheep, high prolificacy sheep breeds in China [24], than our findings of Ossimi and Rahmani.

In our explanation, the two tight linked SNPs in the GDF9 gene (A > G and G > A) were found to be related to litter size, ewe lambing weight, ewe pre-mating weight, DRIL and lamb birth weight in Rahmani and Ossimi sheep. Concerning Rahmani sheep, the heterozygous haplotype (AGGA) was significantly higher in ewe lambing weight and litter size than homozygous one (AAGG). So that, AGGA haplotype can be more hopeful marker for improving ewe lambing weight and litter size than homozygous one (AAGG) regardless of the lambing season. But, the haplotype AAGG can be more reliable marker than AGGA for ewe pre-mating weight and lamb birth weight. Regarding the interaction of GDF9 haplotype and lambing season of ewe, the haplotype AGGA in winter lambing season may be the most suitable for ewe pre-mating weight, litter size and lamb birth weight. The haplotype AGGA in the spring lambing season is more reliable indicator for selection of heavier ewe lambing weight and the shortest period of DRIL. In contrast, AGGA in autumn is not a desirable interaction for lamb birth weight and all the studied traits.

In our findings for Ossimi sheep, the AGGA haplotype was significantly higher than AAGG in ewe pre-mating weight, ewe lambing weight and lamb birth weight. So, the heterozygous haplotype can be more suitable for improving ewe pre-mating weight, ewe lambing weight and lamb birth weight. With respect to the interaction of GDF9 haplotype and lambing season of ewe, the haplotype AGGA in spring lambing season was more reliable marker for heavier ewe pre-mating weight, ewe lambing weight, more litter size and the shortest DRIL period. Also, the heterozygous AGGA in spring and winter is more suitable haplotype for lamb birth weight. The above mentioned explanations confirmed that sheep litter size is determined by genetics and the environmental factors [3]. The observed SNPs in GDF9 can be used as bimolecular markers in selection for the acquired lamb gender in Ossimi sheep. This is because ewes with homozygosity in GDF9 gave birth of more males but for high probability of females in the newborn, you may select heterozygous ones. However, GDF9 genotyping could not be used as a marker for the survival rate of newborn lambs in both Rahmani and Ossimi sheep as no significant differences between the two GDF9 haplotypes in the survival rate of new born lambs.

Previous studies concluded that GDF9 mutations are highly related to litter size in many sheep breeds [24, 25, 27, 31, 32]. Among them, the G >A is called (V332I SNPs) FecGV mutation. The FecGV can increase ovulation rate and so the high rate of twinning in heterozygote ewes than in homozygote ewes in different sheep breeds [20, 24, 25, 31]. These explanations agree with our findings on the twinning rate in both Rahmani and Ossimi breeds. Moreover, Hossain, Suma [33] reported a GDF9 SNP was associated with abdominal size in indigenous sheep. On the other hand, homozygous genotyped ewes had significantly larger litter sizes than heterozygous for Garut sheep in Indonesia [30]. In some cases, ewes with the homozygous mutant of FecGV are infertile due to ovarian and uterine dysplasia [34, 35]. In contrast to our findings, other researchers have found no significant association between those GDF9 mutations and the numbers of newborn lambs in many sheep breeds [21, 36–38]. Besides, no SNPs in the GDF9 gene were detected in the Shal sheep breed [39].

No previous studies discussed the relationship between GDF9 gene polymorphisms and pre-mating weight, lambing weight, lamb gender, lamb birth weight and lamb survival rate. Thus, this study is a novel and so, has an important place in this respect.

## Conclusion

The two observed SNPs (A > G and G > A) in GDF9 gene included with the lambing season probably affect female reproductive traits in Ossimi and Rahmani sheep. The AGGA haplotype of GDF9 can be a potential molecular marker for better pre-mating weight, lambing weight, litter size, DRIL period and lamb birth weight in Ossimi and Rahmani sheep. Moreover, the investigated SNPs can be biomarkers for the lamb gender in Ossimi sheep. Further studies on larger numbers of animals in different sheep populations are required to utilize SNPs in GDF9 gene as genetic markers associated with female reproductive traits in Ossimi and Rahmani sheep.

## Methods

### Animals and samples

The current investigation was conducted on 231 ewes aged 5–7 years from two Egyptian sheep breeds (144 Ossimi and 87 Rahmani). The production and fertility data were recorded for parities in three lambing seasons: winter, spring and autumn (fall). The animals raised in north Egypt belong to farms of Animal Production Research Institute of the Agriculture Research Centre, Ministry of agriculture. The animals were clinically free of diseases and subjected to the same management system. They were maintained under the regular farm feeding program that is composed of 4 Kg clover and hay and 0.25 Kg concentrates per head daily. Blood samples were collected from Jugular veins for each ewe in sterile tubes containing the EDTA as an anticoagulant for DNA extraction. The tubes were held and transported in a box containing ice to the Department of Animal Reproduction in the National Research Centre where preserved in a deep freezer − 20 until DNA isolation. The reproductive performance was evaluated as the pre-mating weight, lambing weight of ewe, litter size, DRIL (days from rams’ introduction into the flock till ewe lambing), lamb birth weight, lamb gender and survival rate of newborn lambs. In each breed, the ewes were categorized according to their observed genotypes into two groups and according to the interaction of genotype and season into six groups.

### DNA extraction

The total genomic DNA was extracted from whole blood samples of ewes by FavoprepTM Blood Genomic DNA Extraction Kits (Cat. No. FABGK 001, FAVORGEN Biotech Corp, Taiwan). The extraction protocol was conducted according to the guidelines and instructions of the kit producers. The DNA elution was held in 50 µl of preheated elution buffer supplied by the kits. DNA samples were subjected to 0.7% agarose gel electrophoresis on in 1× TBE buffer containing ethidium bromide (0.5 µg/ml). The electrophoresis was performed and then inspected by an ultra-violet transilluminator and Gel documentation system (Biorad, USA). After extraction, DNA concentration and purity determination for each sample were measured by a Nanodrop 1000 spectrophotometer (Thermo Scientific, USA) at an optical density of 260/280 nm ratio. After that, 50 ng/µl working concentration was prepared from each DNA sample for conducting polymerase chain reactions (PCR).

### PCR amplification for GDF9 gene

To amplify a fragment of 274 bp in exon 2 of the GDF9 gene through PCR technique, two primers were prepared using the online tool primer3 software.

Version 4.1.0 (http://primer3.ut.ee/) based on the ovis aries GDF9 gene sequence available in the GenBank (from nucleotide 3817 to 4090 in sequence ID: AF078545.2). The sequences of the primers used were F: GAAGAAGCTGCTGAGGGTGT R: GAGAGCCATACCGATGTCCG (Willofort Co., UK). PCR amplifications were carried out as previously performed [40] with slight modifications. Briefly, PCR reactions were performed in 25 µL final volume contained 12.5 µL of 2x cosmo PCR master mixes (Cat. W1020300X, Willofort Co., UK), 2 µL (10 µM) of each (F and R) primer, 7.5 µL nuclease-free water and 1 µL of each DNA sample. The program of PCR was performed through SimpliAmp Thermal Cycler (Applied Biosystems, USA) as an initial denaturation at 95 °C for 3 min. then 29 cycles of denaturation at 95 °C for 30 s., annealing at 57 °C for 30 s., then an extension at 72 °C for 30 s. and a final extension at 72 °C for 3 min.

### Agarose gel electrophoresis

The electrophoresis was performed to check the amplification of the targeted fragment of the GDF9 gene. The PCR products were separated via electrophoresis on 1.5% agarose gel in 1× TBE buffer which contained 0.5 µg/ml ethidium bromide. About 5 µl of PCR product was loaded into the agarose gel and then a 120 volts electrophoresis was run for 45 min. The gel was visualized and imaged using Gel documentation system (Biorad, USA) to inspect PCR bands.

### Single‑strand conformational polymorphism (SSCP)

SSCP analysis was performed on the PCR products (274 bp) of the GDF9 gene for 231 ewes to investigate possible variations in that fragment in the studied populations. PCR-SSCP technique was employed relevant to previous work [41] with some modifications. Briefly, 4 µl PCR products aliquots were added to 12 µl of SSCP denaturing solution (95% formamide, 10 mMNaOH, 0.05% xylene cyanol, 0.025% bromophenol blue and 20 mM EDTA mixed by vortexing and then storing at − 20 °C) and 8 µl distilled water. The cocktail was incubated at 95 °C for 7 min and then rapidly chilled on ice for 10 min. The denatured PCR products were loaded on 10% PAGE gel (acrylamide and bis-acrylamide ratio, 29:1) containing Tetramethylethylene-diamine and ammonium persulphate. Electrophoresis was performed using vertical electrophoresis (Cleaver, UK) contained 0.5× TBE buffer for 14 h (30 mA and 120 V) at 4 c. The gels were stained with 0.5 µg/ml ethidium bromide and visualized using Gel documentation system (Biorad, USA).

### Nucleotide sequencing

The PCR fragments of the GDF9 gene (274 bp) produced different SSCP band patterns were purified using GeneJET PCR Purification Kit (Thermo Scientific, USA, product #K0701) following the kits instructions. Then the purified PCR products were subjected to electrophoresis in 2% agarose gel using 1× TBE running buffer containing 0.5 µg/ml ethidium bromide and visualized by ultra-violet transilluminator before sequencing to confirm the presence of specified PCR bands of GDF9 gene. Then purified PCR samples were utilized to bidirectional (forward and reverse) Sanger sequencing in GATC Biotech Inc., Cologne, Germany according to the running protocol of the company. After that, Codon code Aligner software has been used for multiple sequence alignment to investigate SNPs. The sequence of GDF9 gene of our work was further compared with the sequences in the GenBank using NCBI/BLAST/blastn suite.

### Statistical analysis

The frequencies of the GDF9 genotypes in Ossimi and Rahmani sheep and nonparametric data like gender of lambs and survival rate of lambs were statistically analyzed by SPSS Statistics software version 25 through Chi-square test. The probability value was reported statistically significant when it is less than 0.05. All the data were statistically analyzed through two-way analysis of variance by using general linear model (GLM) through xlstat software version 2019. The main effects in GLM were breeding season and Genotype. Traits analyzed were: ewe pre-mating weight, ewe lambing weight, litter size, DRIL and lamb birth weight.

The model used was $\text{Yijk}=\upmu+\text{Li}+\text{Sj}+\text{LSij}+\text{eijk}$.

Where:

$\text{Yijk}$ : The kth observation of the jth Genotype within the ith lambing season.

$\text{M}$: The overall mean.

$\text{Li}$: The effect of the ith lambing season.

$\text{Sj}$: The effect of the jth Genotype.

$\text{Lsij}$: The interaction between the ith lambing season and the jth Genotype.

$$\text{Eijk}$$: Random error.

All the data were presented as least square means (LSM) ± standard errors of mean (SEM). Mean values were separated when significance was present using Duncan’s test [42]. Significance level was set at 5%.

## Supplementary Information

Supplementary Material 1.

Supplementary Material 2.

Supplementary Material 3.

Supplementary Material 4.

## Data Availability

All the data are available from the corresponding author upon reasonable request. The sequence data were submitted to the GenBank NCBI genomic database and got accession number; **PV089625.** The information are publicly available in the link; https://www.ncbi.nlm.nih.gov/nuccore/PV089625.1.
