# Supplementary figures and images for: Investigation of SNPs in GDF9 gene and their relationship with some reproductive traits of Ossimi and Rahmani sheep in different lambing seasons
Source: BMC Vet Res. 2025 Dec 2;21:704. doi: 10.1186/s12917-025-05145-5 (PMC12702159; doi:10.1186/s12917-025-05145-5)

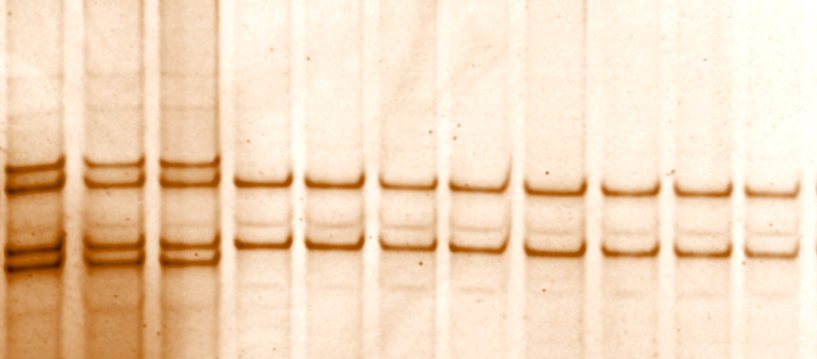

Supplement: Supplementary file 2 — Supplementary Material 2. [file 12917_2025_5145_MOESM2_ESM.zip › another fig 2 SSCP.tif]

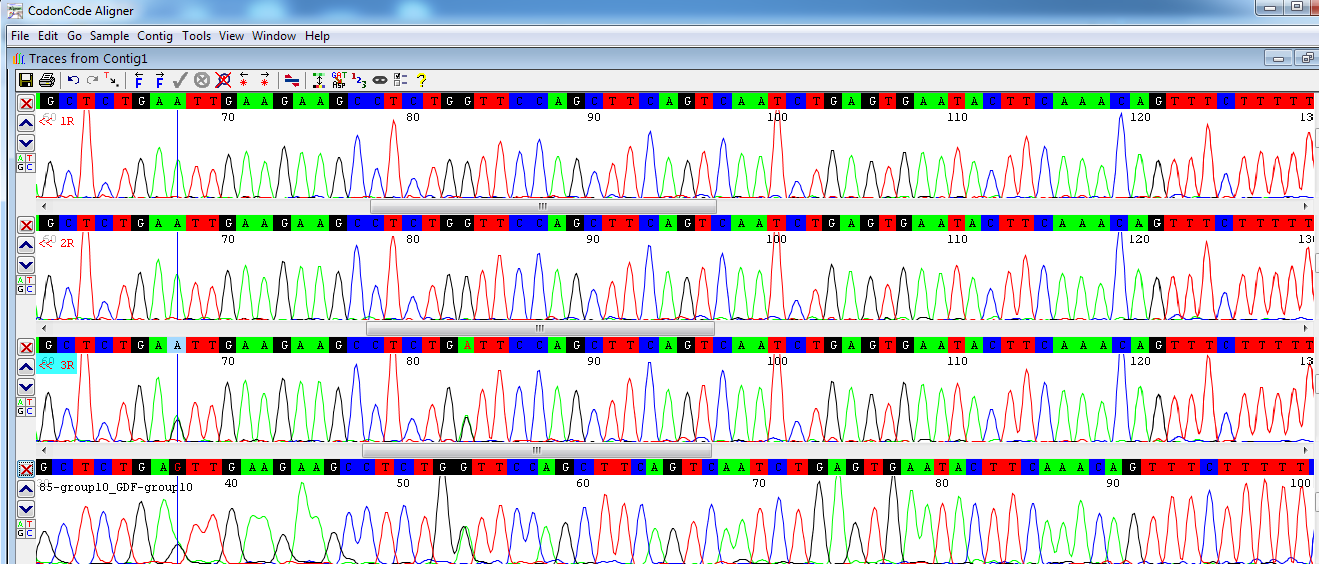

Supplement: Supplementary file 2 — Supplementary Material 2. [file 12917_2025_5145_MOESM2_ESM.zip › another fig 3 GDF9 partial sequence1.tif]

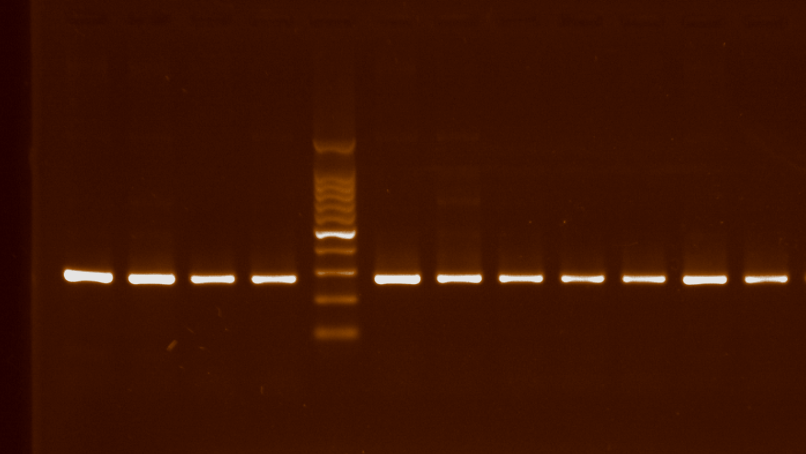

Supplement: Supplementary file 2 — Supplementary Material 2. [file 12917_2025_5145_MOESM2_ESM.zip › another fig. 1 PCR.tif]

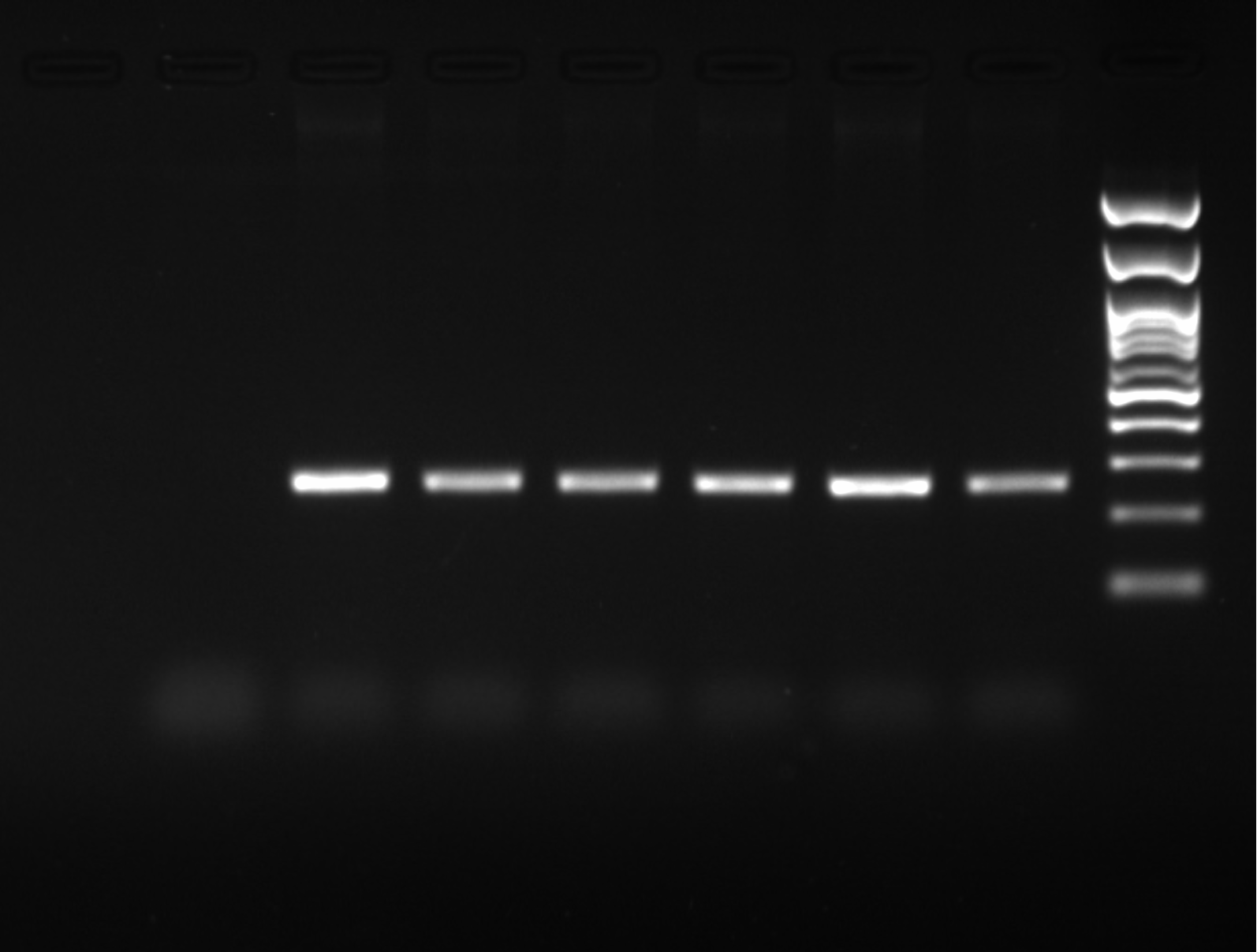

Supplement: Supplementary file 2 — Supplementary Material 2. [file 12917_2025_5145_MOESM2_ESM.zip › Fig. 1 GDF9 274 bp PCR product.tif]

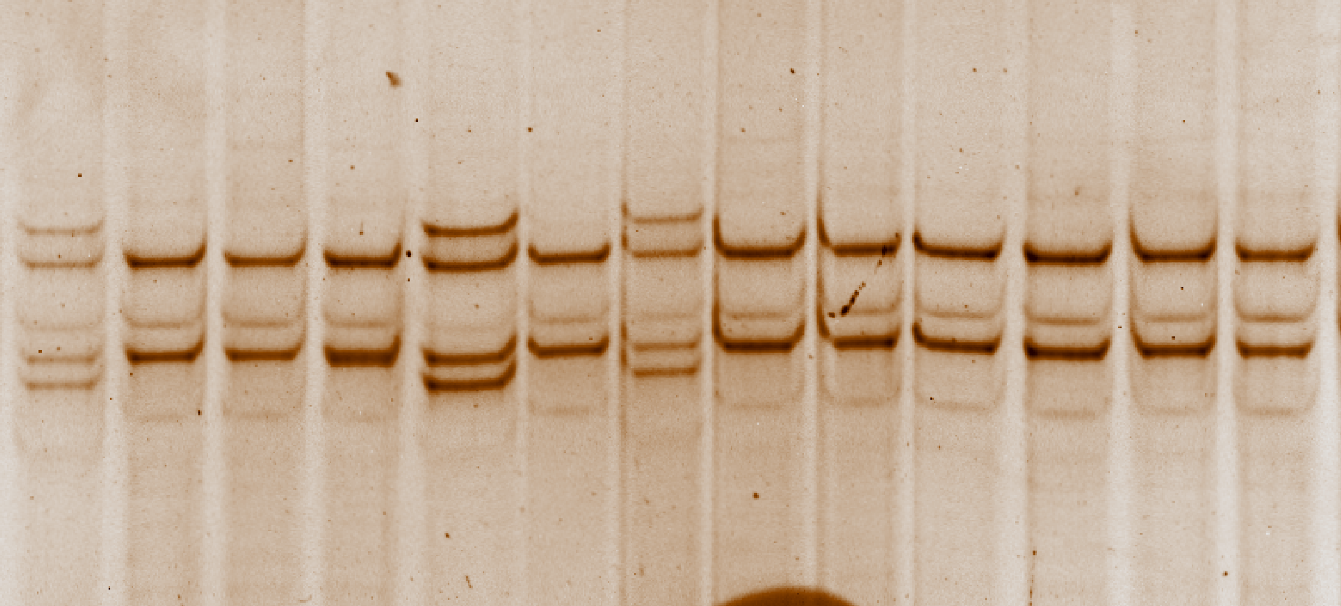

Supplement: Supplementary file 2 — Supplementary Material 2. [file 12917_2025_5145_MOESM2_ESM.zip › Fig. 2 SSCP GDF9 gene.tif]

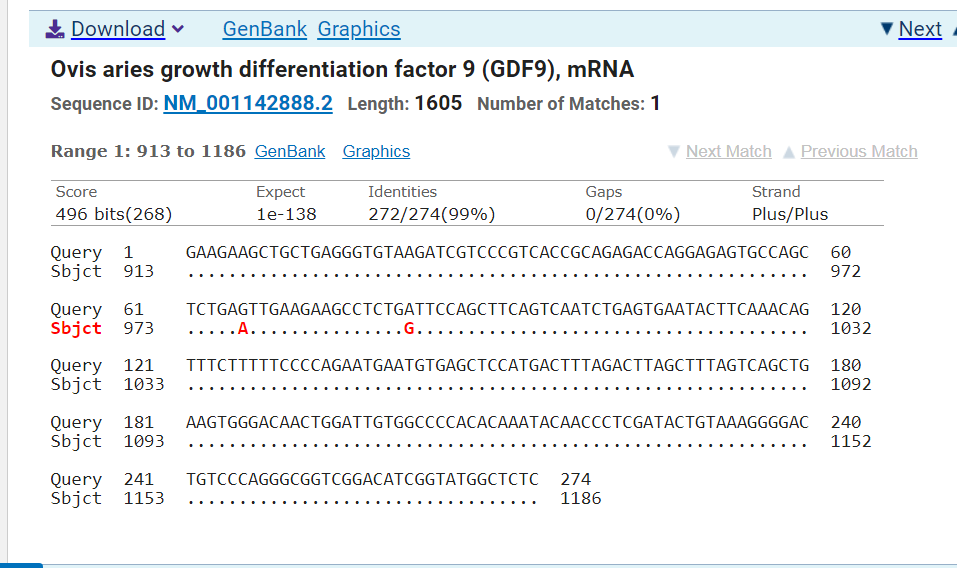

Supplement: Supplementary file 2 — Supplementary Material 2. [file 12917_2025_5145_MOESM2_ESM.zip › Fig. 4 gdf9 alignment.tif]

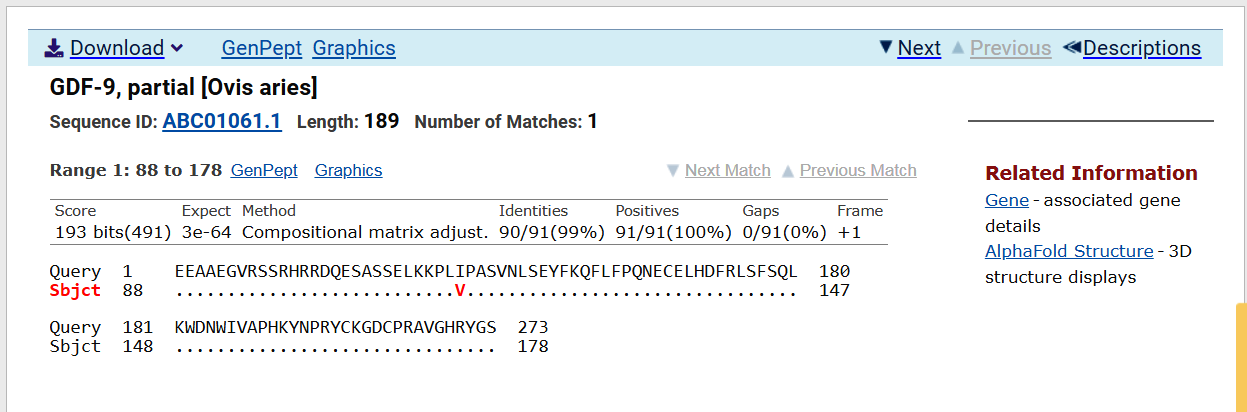

Supplement: Supplementary file 2 — Supplementary Material 2. [file 12917_2025_5145_MOESM2_ESM.zip › Fig. 5 The amino acid sequence alignment GDF9.tif]

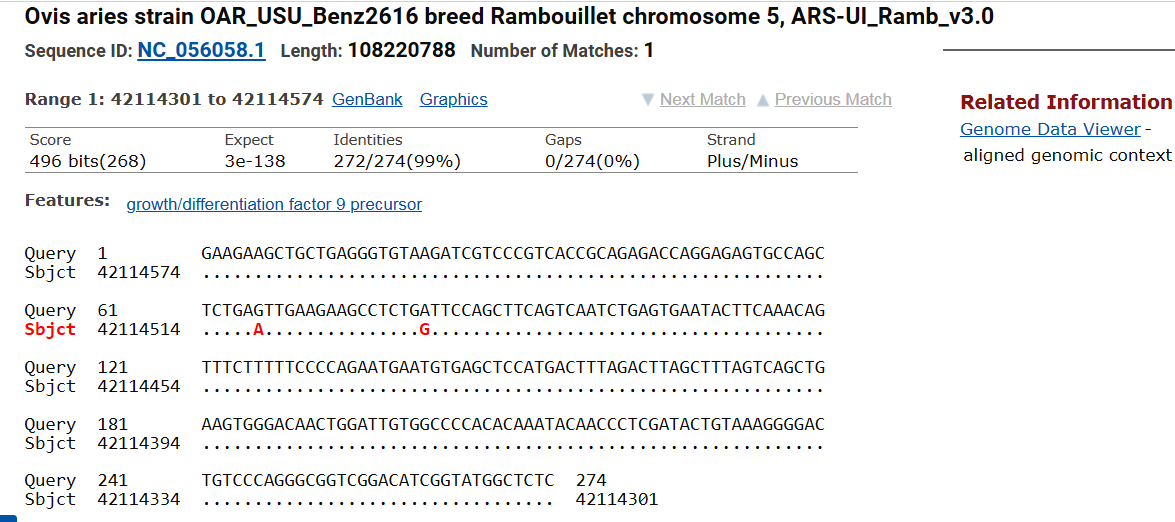

Supplement: Supplementary file 2 — Supplementary Material 2. [file 12917_2025_5145_MOESM2_ESM.zip › GDF in Genome (ARS-UI_Ramb_v3.0 reference assembly GCF_016772045.2.tif]

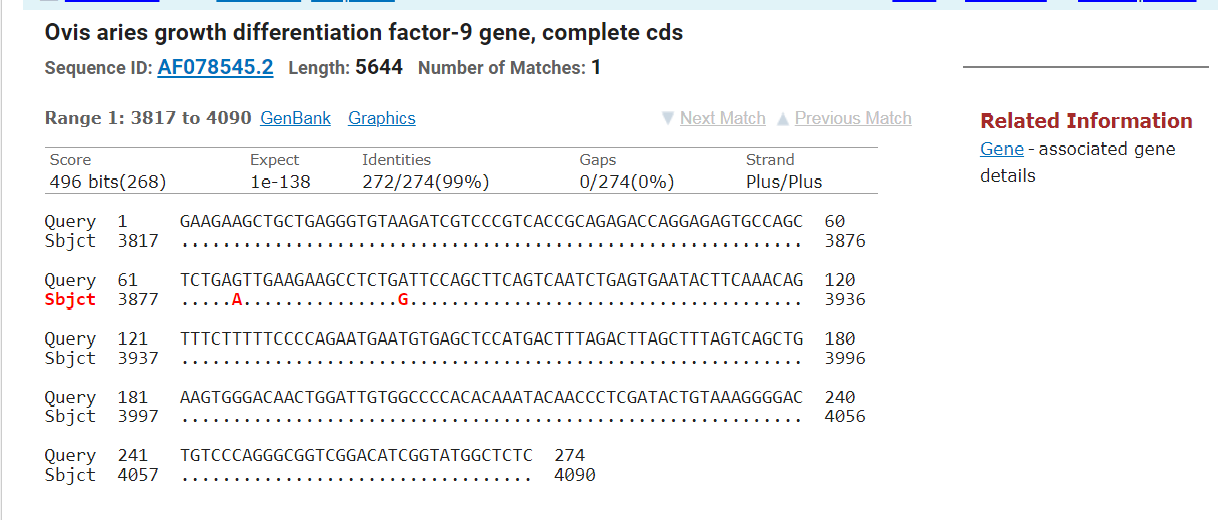

Supplement: Supplementary file 2 — Supplementary Material 2. [file 12917_2025_5145_MOESM2_ESM.zip › other fig 4 GDF9 p2.tif]

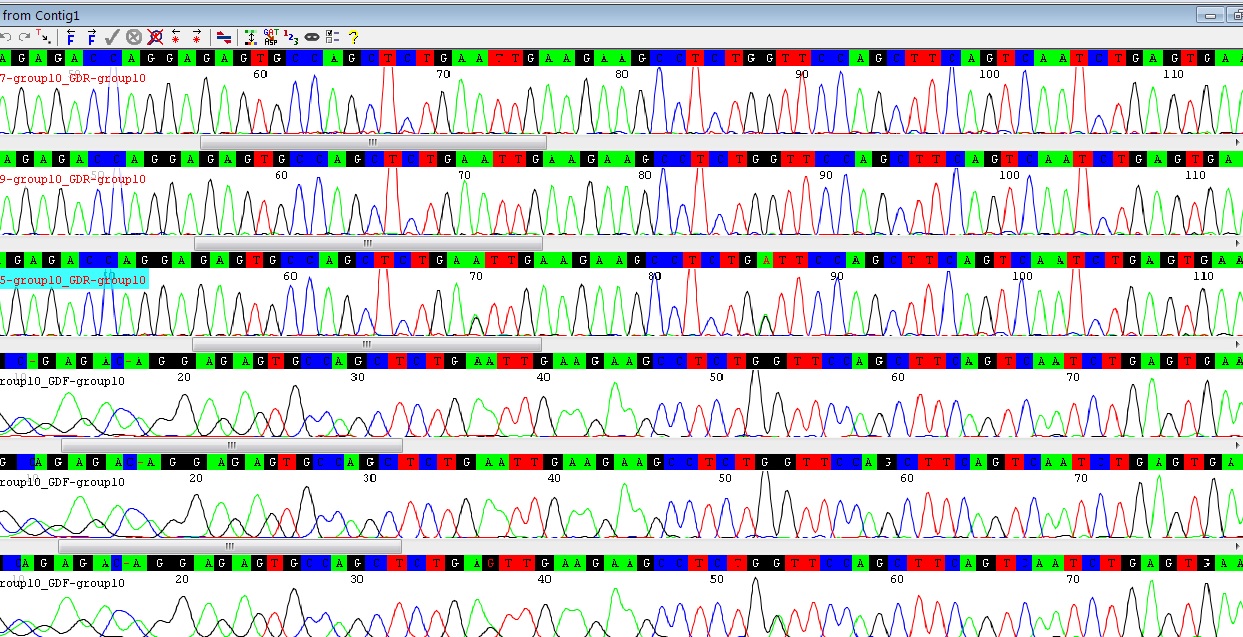

Supplement: Supplementary file 3 — Supplementary Material 3. [file 12917_2025_5145_MOESM3_ESM.jpg]
