# Supplementary material for: Investigation of SNPs in GDF9 gene and their relationship with some reproductive traits of Ossimi and Rahmani sheep in different lambing seasons
Source: BMC Vet Res. 2025 Dec 2;21:704. doi: 10.1186/s12917-025-05145-5 (PMC12702159; doi:10.1186/s12917-025-05145-5)
Supplement: Supplementary file 4 — Supplementary Material 4. [file 12917_2025_5145_MOESM4_ESM.pdf]

```
LOCUS       1                        274 bp    DNA     linear   MAM 10-
FEB-2025
DEFINITION  Ovis aries Growth differentiation factor 9 (GDF9) gene 274
bp.
ACCESSION   1
VERSION
KEYWORDS    .
SOURCE      Ovis aries (sheep)
  ORGANISM  Ovis aries
            Eukaryota; Metazoa; Chordata; Craniata; Vertebrata;
Euteleostomi;
            Mammalia; Eutheria; Laurasiatheria; Artiodactyla; Ruminantia;
            Pecora; Bovidae; Caprinae; Ovis.
REFERENCE   1  (bases 1 to 274)
  AUTHORS   Hasanain,M.H., Sosa,A.S.A., Darwish,H.R.H., Sakr,A.M. and
            Shedeed,S.M.
  TITLE     Direct Submission
  JOURNAL   Submitted (10-FEB-2025) Animal Reproduction & A.I., National
            Research Centre, Tahreer street, Cairo, Giza 12622, Egypt
COMMENT     Bankit Comment: ALT EMAIL:mh.hasanain@nrc.sci.eg
            Bankit Comment: TOTAL # OF SEQS:1

            ##Assembly-Data-START##
            Sequencing Technology :: Sanger dideoxy sequencing
            ##Assembly-Data-END##
FEATURES             Location/Qualifiers
     source          1..274
                     /organism="Ovis aries"
                     /mol_type="genomic DNA"
                     /isolation_source="blood"
                     /host="Sheep"
                     /db_xref="taxon:9940"
                     /chromosome="5"
                     /haplotype="AGGA"
                     /sex="Female"
                     /dev_stage="adult"
                     /country="Egypt"
                     /collected_by="authors"
                     /breed="Ossimi and Rahmani"
                     /note="extrachromosomal"
     gene            <1..274
                     /gene="GDF9"
                     /allele="AGGA"
     CDS             <1..274
                     /gene="GDF9"
                     /allele="AGGA"
                     /codon_start=1
                     /product="GDF9 protein"

/translation="EEAAEGVRSSRHRRDQESASSELKKPLIPASVNLSEYFKQFLFP
                     QNECELHDFRLSFSQLKWDNWIVAPHKYNPRYCKGDCPRAVGHRYGS"
BASE COUNT       71 a     66 c     71 g     66 t
ORIGIN
```

```
        1 gaagaagctg ctgagggtgt aagatcgtcc cgtcaccgca gagaccagga
gagtgccagc
       61 tctgagttga agaagcctct gattccagct tcagtcaatc tgagtgaata
cttcaaacag
      121 tttctttttc cccagaatga atgtgagctc catgacttta gacttagctt
tagtcagctg
      181 aagtgggaca actggattgt ggccccacac aaatacaacc ctcgatactg
taaaggggac
      241 tgtcccaggg cggtcggaca tcggtatggc tctc
//
```
